# Dried Blood Specimens as an Alternative Specimen for Immune Response Monitoring During HIV Infection: A Proof of Concept and Simple Method in a Pediatric Cohort

**DOI:** 10.3389/fmed.2021.678850

**Published:** 2021-06-15

**Authors:** Marina Rubio-Garrido, José Avendaño-Ortiz, Adolphe Ndarabu, Carolina Rubio, Gabriel Reina, Eduardo López-Collazo, África Holguín

**Affiliations:** ^1^HIV-1 Molecular Epidemiology Laboratory, Microbiology Department, Ramón y Cajal University Hospital- Instituto Ramón y Cajal para la Investigación Sanitaria (IRYCIS) and Centro de Investigación Biomédica en Red de Epidemiología y Salud Pública - Red de Investigación Traslacional en Infectología Pediátrica (CIBERESP-RITIP), Madrid, Spain; ^2^Innate Immunity Group, Hospital La Paz Institute for Health Research, La Paz University Hospital, Madrid, Spain; ^3^Tumor Immunology Laboratory and Innate Immunity Group, Hospital La Paz Institute for Health Research, La Paz University Hospital, Madrid, Spain; ^4^Monkole Hospital, Kinshasa, Democratic Republic of Congo; ^5^University Clinic of Navarra, Pamplona, Spain

**Keywords:** dried blood specimen, human immunodeficiency virus, immune markers, inflammation, RNA expression, children

## Abstract

Programs to prevent mother-to-child HIV transmission do not reduce the number of infants exposed during pregnancy and breastfeeding. HIV-exposed but uninfected children (HEU) present higher risk of morbidity and mortality than HIV-unexposed and uninfected children (UU). In this line, the study of immune biomarkers in HIV could improve prediction of disease progression, allowing to diminish comorbidity risk. Dried blood specimens (DBS) are an alternative to serum for collecting and transporting samples in countries with limited infrastructure and especially interesting for groups such as pediatrics, where obtaining a high sample volume is challenging. This study explores the usefulness of DBS for immune profile monitoring in samples from 30 children under clinical follow-up in Kinshasa: 10 HIV-infected (HIV+), 10 HEU, and 10 UU. We have measured the gene expression levels of 12 immune and inflammatory markers (CD14, IL-6, TNFα, HVEM, B7.1, HIF-1α, Siglec-10, IRAK-M, CD163, B7H5, PD-L1, and Galectin-9) in DBS samples by reverse transcription of total RNA and RT-qPCR. Principal component analysis, Kruskal–Wallis test, and Mann–Whitney test were performed in order to study group differences. HIV+ children presented significantly higher levels of seven biomarkers (CD14, IL-6 HVEM, B7.1, Siglec-10, HIF-1α, and CD163) than the UU group. In HEU, we found seven biomarkers significantly elevated (CD14, IL-6, HVEM, B7.1, Siglec-10, HIF-1α, and IRAK-M) vs. UU. Six biomarkers (CD14, IL-6, HVEM, B7.1, Siglec-10, and HIF-1α) showed a significantly higher expression in both HIV+ and HEU vs. UU, with HVEM and CD14 being significantly overexpressed among HIV+ vs. HEU. Our data reveal the utility of DBS for immune response monitoring. Moreover, significant differences in specific biomarker expression across groups strongly suggest the effect of HIV infection and/or HIV exposure on these immune biomarkers' expressions.

## Introduction

Among the 38 million people living with human immunodeficiency virus (HIV), more than half are women and almost 2 million are children below the age of 15 (1). An early diagnosis and correct HIV treatment monitoring with routine viral load testing in infected adults and children is required to control HIV epidemics worldwide (2). The implementation of programs to prevent mother-to-child HIV transmission (MTCT) reduces the number of newly infected children. However, high maternal HIV incidence and deficient HIV testing and viremia control for pregnant and postpartum women substantially contribute to viral transmission and to HIV exposure in infants during pregnancy and breastfeeding in environments with a high HIV burden. In 2019, 110,000 children (aged 0–14 years) acquired HIV infection in the 21 focus countries (3). One of these countries is the Democratic Republic of Congo (DRC), with 0.8% HIV prevalence [0.60.9], (1, 4), high rates of MTCT (5), low antenatal care coverage, and having less than half of the pregnant women living with HIV receiving antiretroviral therapy (3). In the DRC, about 51,000 children are living with HIV, mainly due to the high MTCT rate of 27.1% [24.4–30], in addition to 310,000 HIV-exposed but born uninfected children (HEU), according to the estimation of the most recent Joint United Nations Programme on HIVAIDS (UNAIDS) (1).

HEU present 3.9 and 2.0-fold higher mortality rate than HIV-unexposed (born to mothers infected with HIV) and uninfected children (UU) during the first and second year of life, respectively (6). Mortality is most commonly due to acute respiratory infections, diarrhea/dysentery, malnutrition, sepsis, or meningitis. HEU are more likely to be premature and smaller than normal for their gestational age than UU, have more postnatal growth failure, and have 50% more hospitalizations in the neonatal period and 30% more hospital visits during infancy, particularly for skin infections, lower respiratory tract infections, and oral thrush (6). Lower rates of breastfeeding in exposed infants can reduce their protection from HIV and other infections, with an increased risk of death from malnutrition, diarrhea, and pneumonia if the infants are not exclusively breastfed (7). In addition, reduced care due to parental illness or death can also increase the exposure to other infections among HEU (8). Nonetheless, despite the public health importance of the great number of HEU involved in the epidemic, health supervision specifically addressing the HEU population is scarce worldwide and has been largely neglected (8).

The increased risk of infectious morbidity, mortality, and growth failure observed among HEU (8) is due to greater immune activation and inflammation (6, 9–12), with lower T cell functionality and higher monocyte activation in HEU vs. UU (11, 13). Expression of immune checkpoints, regulators of T cell immune activities in response to invading pathogens (14), and of other immunological biomarkers can be altered during HIV infection despite viremia control or undetectable viral load (15). These checkpoints can be used as biomarkers for monitoring HIV disease progression and therapeutic response in individuals infected with HIV (14), just as they do in other chronic infectious diseases and cancer (16, 17). These can also predict the risk of comorbidities associated with chronic inflammation (18). However, immune and inflammatory markers usually need to be measured in fresh blood or plasma frozen samples, making their study difficult in resource-poor countries (19).

Dried blood specimens (DBS) use facilitates blood samples collection, storage, and transport to centralized facilities for HIV diagnosis and monitoring in environments with limited infrastructure, as well as when large blood volumes are not available, such as in neonates and in children with low weight (20). Studies reporting different expression levels of immune biomarkers in HEU are scarce and are limited to a few biomarkers, mainly in plasma (10, 11, 13, 21). This pilot study provides pioneer data on the gene expression of 12 immune biomarkers, including checkpoint and inflammatory markers, obtained by reverse transcription-quantitative polymerase chain reaction (RT-qPCR), using DBS in three groups of participants: HIV-infected (HIV+), HEU, and UU, in a pediatric cohort from the DRC. The main objective was to evaluate the usefulness of DBS samples as an alternative to the plasma or serum samples. In addition, we analyzed the influence of HIV infection and HIV exposure on the immune status of three groups of children (HIV+, HEU, and UU). To our knowledge, this is the first study to use dried blood (not plasma/serum) to measure immune biomarkers expression in HIV-exposed children.

## Methods

### Study Design and Sample Collection

The study was conducted in accordance with the ethical guidelines of the 1975 Declaration of Helsinki and was approved by La Paz Hospital Ethics Committee. An informed consent was obtained from parents, legally acceptable representatives, or guardians of participants' statements. The STROBE statement for observational studies is provided in the Supplementary Material for more details in the study design.

DBS were collected from 85 pediatric participants under clinical follow-up in Monkole Hospital in Kinshasa (DRC) from April to November 2016. We checked the HIV status of them all and selected 10 patients with HIV infection (HIV+), 10 patients from confirmed HIV+ mother (exposed) but uninfected (HEU), and 10 uninfected and unexposed children (UU). Participants had a median [IQR] age of 12.2 [10.7–15.3], 3 [0–8.5], and 10.5 [8.3–12] years old, respectively. This research is considered a case-control study in which the UU group is the control group and its results were compared with HEU and HIV+ groups. DBS were prepared by spotting venous blood collected by venopuncture in EDTA anticoagulant tubes with a 70-μl micropipette into each spot on a Whatman 903 Protein Saver Card (Schleicher & Schuell, Dassel, Germany).

### HIV Diagnosis and Viremia Quantification

HIV diagnosis was firstly performed in DRC using rapid serological tests: Determine™ HIV-1/2 Ag/Ab (Alere), Double-Check Gold HIV 1&2 (Orgenics), and Uni-Gold HIV (Trinity Biotech) from 18 months old and by Biomerieux 4th-generation immunoassay VIDAS® HIV Duo Ultra or, in exceptional cases, by molecular Abbott real-time HIV-1 Qualitative in infants under the age of 18 months old. In Madrid, Spain, HIV serological status in the 85 children was confirmed with the BioRad Geenius^TM^ HIV-1/2 confirmatory assay using one DBS spot per patient, as previously reported (22). All HIV seropositive and undetermined pediatric DBS by Geenius were then tested by molecular POC test Cepheid Xpert Qual (Xpert Qual), which provides a binary “detected”/“not detected” result (23). HIV-1 viremia was quantified using the COBAS® AmpliPrep/COBAS® TaqMan® HIV-1 Test v2.0 (Roche VL) (24) in all HIV+DBS, based on real-time amplification of HIV genome. All assays were performed using one spot eluted in Roche SPEX buffer as lysis buffer to elute the DBS spots, according to manufacturer's instructions. We provided the number of HIV-1 RNA copies per plasma milliliter after considering 39% hematocrit as the standard value for children (25).

### Tested Immune Biomarkers

We analyzed the levels of 12 immune biomarkers: B7.1 (membrane protein presents in activated antigen-presenting cells) (26); B7H5 (V-domain Ig suppressor of T cell activation) (27); IRAK-M (interleukin-1 receptor associated kinase) (26), associated with inflammatory bowel diseases (28); Galectin-9 (glycan-binding protein) (29); Siglec-10 (sialic acid-binding Ig-like lectin 10) (30); HIF-1α (hypoxia-inducible factor 1-alpha, a transcription factor) (26); HVEM (herpes virus entry mediator) (29); CD14 (co-receptor for lipopolysaccharide released from monocytes upon activation) (31); CD163 (marker of monocyte/macrophage activated cells) (31); PD-L1 (programmed death-ligand 1) (26, 29); IL-6 (B cell stimulatory factor 2) (32, 33); and TFNα (tumor necrosis factor-α, a proinflammatory cytokine) (34, 35). These biomarkers were selected because of their previous reported implication in HIV and other infections/pathologies (26, 29, 31).

### Biomarker Quantification by RT-PCR

**Figure 1:**
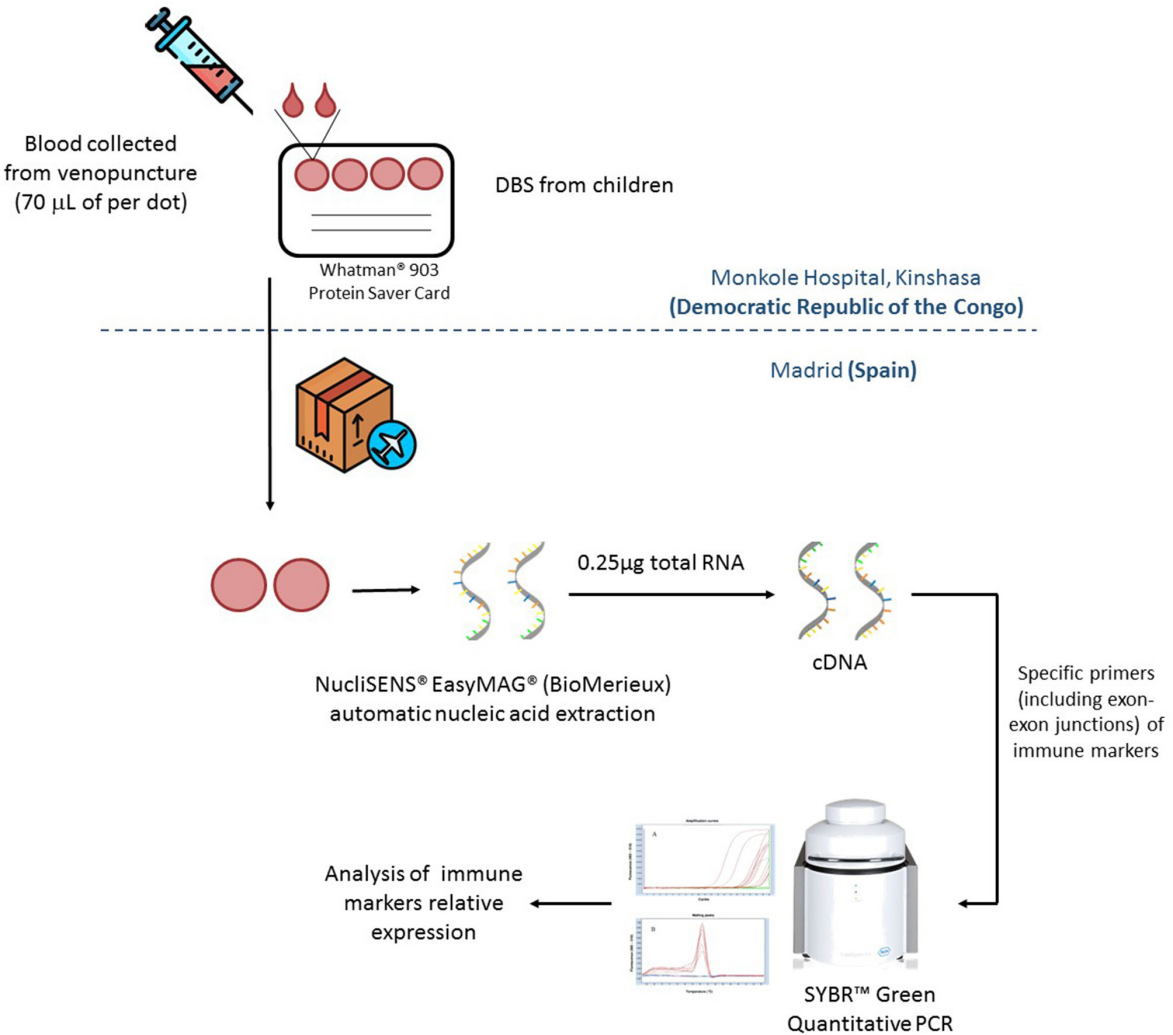
Dried blood spots (DBS) samples from Kinshasa Hospital were send to Madrid in dried ice for their analysis. Once in Madrid, nucleic acids were extracted using two spots (70 μl of dried blood per spot) per patient by NucliSENS® EasyMAG® (BioMerieux). Total mRNA was quantified by Nanodrop and 0.25 μg of total RNA was retrotranscribed to complementary DNA (cDNA) by using High-Capacity cDNA Reverse Transcription Kit (Applied Biosystems). Relative expression of immune markers was quantified by qRT-PCR in a LightCycler ® 480 (Roche).

A brief scheme of the procedure is included in Figure 1. To quantify these immune biomarkers in each patient, nucleic acids were extracted from two DBS spots using the NucliSENS EasyMAG automated platform (BioMerieux). cDNA was synthesized from 0.25 μg of total RNA using the High-Capacity cDNA Reverse Transcription Kit (Applied Biosystems). Reverse transcription quantitative polymerase chain reactions (RT-qPCRs) were performed using the QuantiMix Easy SYG Kit (Biotools), according to the manufacturer's instructions. Gene expression levels were analyzed using the LightCycler system (Roche Diagnostics). The cDNA copy number of each gene of interest was determined using a seven-point standard curve. Reactions were run in duplicate and the expression level of the β-ACTIN housekeeping gene was used as internal standard to normalize data in the relative expression of each biomarker. All the primers included exon–exon junctions to avoid genomic DNA contamination and were synthesized by Europhins Genomics. Specific primers for each gene are shown in Supplementary Table 1.

### Statistical Analysis

Principal components of the biomarkers' relative expression data were calculated using the function princomp of R language. The default method performed a principal components analysis on the given numeric data matrix and returned the results as an object of class. The calculation was done by a singular value decomposition of the centered and scaled data matrix. The print method for these objects and the plot method were performed using the packages R ggiplot and ggbiplot. The Kruskal–Wallis non-parametric test was performed for overall comparison of the three groups. After that, the Mann–Whitney *U* test was used to study the differences among groups. The significance level used for the hypothesis contrast tests was set at *P* < 0.05. Statistical analyses were conducted using GraphPad Prism 6.0 software.

## Results

### Study Population

The study's main features of the pediatric population involved are shown in Table 1. Both HIV+ and UU groups presented similar median age at sampling, higher than the HEU group (12 and 10.5 years old vs. 3 years old, respectively). All children with HIV were under antiretroviral treatment, although none presented suppressed viremia at sampling. Among the HEU group, seven had previously received antiretroviral drugs, five as prophylaxis. Four of the HEU group presented maternal anti-HIV antibodies at sampling, all under 18 months of age (from 0 to 4.8 months old).

**Table 1:** Main features of the study cohort.

	**HIV+**	**HEU**	**UU**
Number of children	10	10	10
Male	6	5	5
Median age at sampling in years [IQR]	12 [10.7–15.3]	3 [0–8.5]	10.5 [8.3–12]
Breastfed	5	7	Unknown
Positive confirmatory serological HIV status by Geenius	10	4	0
Positive HIV+ molecular diagnosis by Xpert HIV-1 Qual	10	0	0
Antiretroviral exposure after birth	10	7 (5 prophylaxis)	0
Mean VL (log/ml) (range) by Cobas Roche	4.3 (2.7–5.3)	-	-

*HIV+, HIV-1 infected children; HEU, HIV-exposed but born uninfected children; UU, HIV-unexposed and uninfected children; VL, HIV-1 RNA copies per plasma milliliter (ml); log, logarithm. Geenius, Bio-Rad Geenius^TM^ HIV 1/2 Confirmatory Assay (36); Xpert HIV-1 Qual, Cepheid Xpert HIV-1 Qual (23)*.

### DBS Samples From HIV-Infected Children Are Viable Specimens for Host mRNA Differential Expression Studies

First, we decided to evaluate if DBS samples were robust enough specimens for the analysis of host mRNA expression. Children blood DBS samples were transported from Kinshasa to Spain, where they were analyzed. We obtained a mean ± SD of 0.86 ± 0.27 μg of total RNA per sample after nucleic acid extraction from two blood spots (≈140 μl of blood) of each child. We utilized 0.25 μg for retro transcription to cDNA. The expression of the housekeeping gene β-actin amplified by specific primers including exon–exon junction was used as the housekeeping gene to validate the RT-qPCRs. Detectable levels of β-actin were found in all analyzed samples with a mean Threshold point (Ct) of 22.13 ± 2.11.

### HIV-Infected, -Exposed, and -Unexposed Children Have Differential Immune Marker Profiles

**Figure 2:**
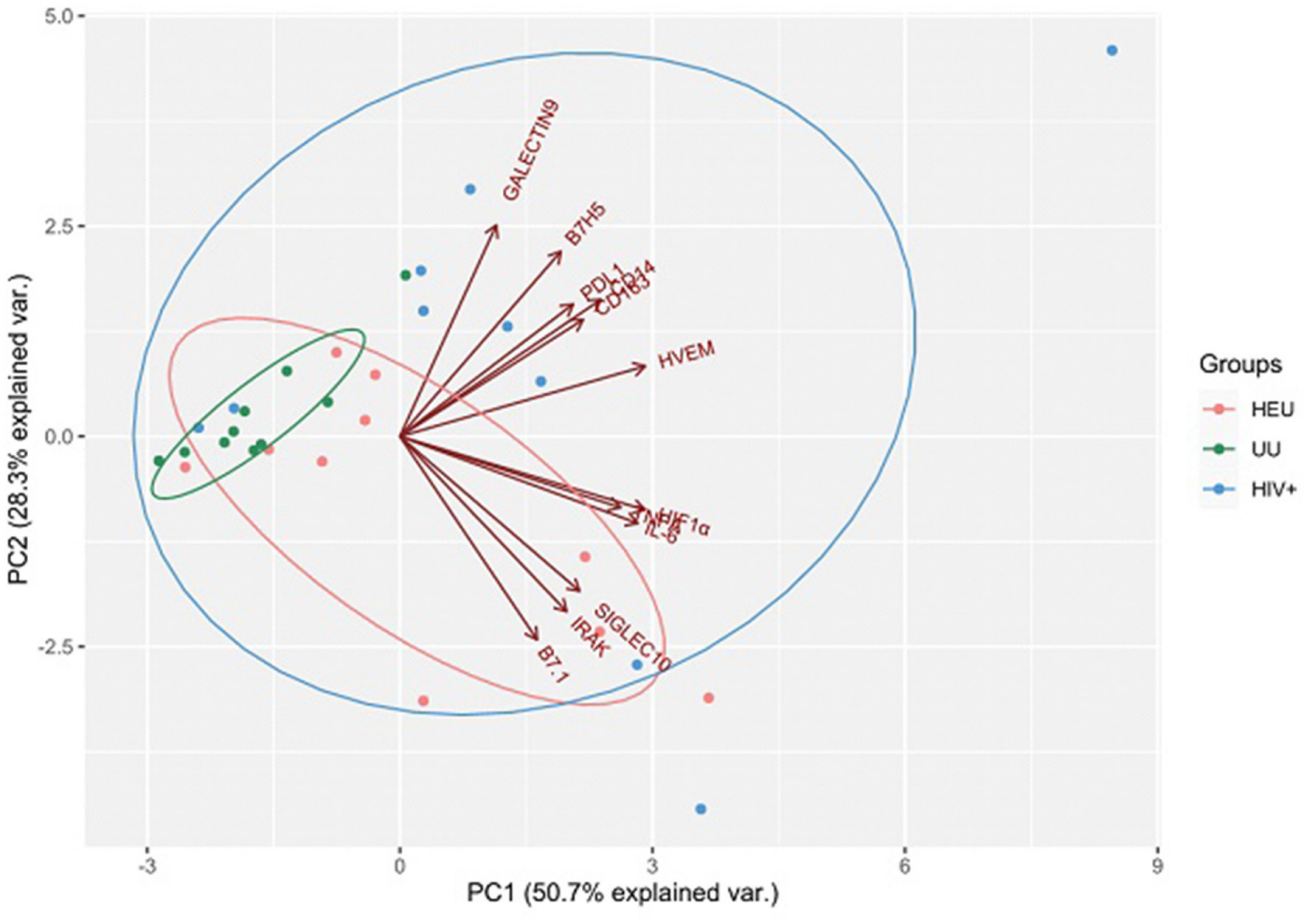
Results are plotted according to the first two principal components accounting for 79% of the total variation (Component 1 = 50.7% and Component 2 = 28.3%). Each dot represents data from a single child sample: HIV-exposed but uninfected (HEU) in orange; HIV-infected (HIV+) in blue; HIV-unexposed and uninfected (UU) in green. The clustering of samples is represented by their respective 95% confidence interval ellipse from centroid. Vectors of each immune marker are represented by red lines.

Once the usefulness of DBS samples was checked, we moved on to analyze the effects of HIV infection and exposure on immune markers, studying overall differences between children subgroups. We selected 12 immune markers including immune checkpoints (CD14, IL-6, TNFα, HVEM, B7.1, HIF-1α, Siglec-10, IRAK-M, CD163, B7H5, PD-L1, and Galectin-9). Some of these had been previously associated to a worse prognosis in HIV and other infectious diseases (26, 37, 38), or related to inflammatory status (IL-6, CD14, CD163, IRAK-M, and TNFα) (12, 21, 39, 40). We first performed a principal component analysis (PCA) for the expression of 10 immune markers in DBS samples from our cohort. The four principal components explained 89.8% of variance (Table 2). The PCA results showed that the immune marker profiles in UU children substantially differed from the HEU and HIV-infected groups (Figure 2).

**Table 2:** Principal component analysis of 10 immune markers from 30 pediatric samples from the study pediatric cohort.

**Principal component**	**1**	**2**	**3**	**4**
**Vector values for the component**				
B7.1	0.21	−0.41	−0.39	0.15
B7-H5	0.24	0.37	0.16	−0.20
CD14	0.30	0.28	−0.20	0.25
CD163	0.28	0.23	−0.34	−0.27
HIF-1α	0.37	−0.15	0.27	−0.06
HVEM	0.37	0.14	−0.13	0.00
IRAK-M	0.25	−0.35	−0.01	−0.11
Galectin-9	0.14	0.42	−0.23	−0.35
PD-L1	0.26	0.27	0.18	0.78
Siglec-10	0.27	−0.31	−0.45	0.09
TNF-α	0.33	−0.14	0.46	−0.17
IL-6	0.36	−0.17	0.29	−0.16
**Percent of total variance explained by the component**	50.6	28.3	7.7	3.2

**Figure 3:**
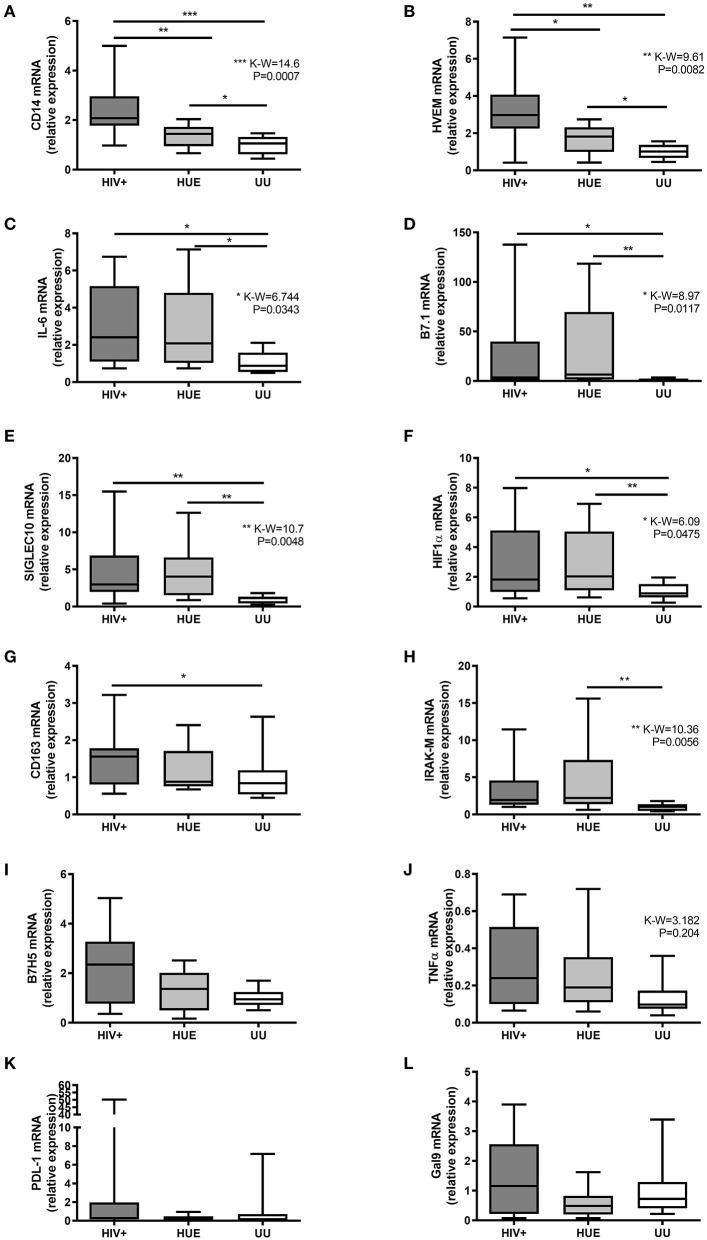
Relative expression by RT-qPCR of CD14 **(A)**, HVEM **(B)**, IL-6 **(C)**, B7.1 **(D)**, Siglec-10 **(E)**, HIF-1α **(F)**, CD163 **(G)**, IRAK-M **(H)**, B7H5 **(I)**, TNFα **(J)**, PD-L1 **(K)**, and Galectin-9 **(L)** genes on mRNA extracted from DBS from a pediatric cohort of DRC. HIV+, HIV-infected children; HEU, HIV-exposed but born uninfected children; UU, HIV-unexposed and uninfected children; P-values and Kruskal-Wallis statistics (K-W) for statistically significant biomarkers are shown. *P < 0.05; **P < 0.01; ***P < 0.001 on Mann-Whitney U-test between groups. Figure presented in IAS Congress 2019, abstract TUPEC444.

We carried on to study the expression profile of each marker separately. We identified how some immune markers increased in children with HIV infection when compared to the HEU and UU groups. HIV+ children presented significantly higher levels of seven of the 12 biomarkers (CD14, HVEM, IL-6, B7.1, Siglec-10, HIF-1α, and CD163) compared to the UU group (Figures 3A–G). The T cell response suppressor B7H5 and the inflammatory cytokine TNFα also exhibited a higher expression in the HIV+ group compared with the UU group, but with no statistical significance (Figures 3I,J). On one hand, six of these biomarkers (CD14, HVEM, IL-6, B7.1, Siglec-10, and HIF-1α) were related to both HIV exposure and infection, presenting significantly higher expression in both the HIV+ and HEU groups than in the UU group (Figures 3A–F). CD14 and HVEM were significantly more expressed in the HIV+ than the HEU (Figures 3A,B), and the expression of IL-6, B7.1, Siglec-10, HIF-1α, CD163, IRAK-M, B7H5, TNFα, PD-L1, and Galectin-9 was similar in the HIV+ and HEU groups (Figures 3C–L).

On the other hand, we found immune markers that increased with HIV exposure. When comparing HEU with UU, seven biomarkers were significantly elevated after HIV exposure (CD14, HVEM, IL-6, B7.1, Siglec-10, HIF-1α, and IRAK-M) (Figures 3A–F,H). IRAK-M was only increased by HIV exposure, presenting significantly higher levels in the HEU vs. UU group (Figure 3H). Note that we found no statistically significant correlations between age and any of the 12 studied immune markers (Supplementary Figure 1).

## Discussion

One of the main challenges today is to improve the diagnosis and monitoring of HIV and other pathogens in countries with limited resources. In this sense, the use of DBS has emerged as a great specimen due to its easier sampling, transport, and storage (20), being extensively used in many countries. In the DRC, DBS have been used for HIV diagnosis, viral load quantification, drug levels and resistance monitoring, HIV subtyping, and seroprevalence studies, among others (41).

For more than a decade, DBS samples have been used to quantify mRNA expression of immune markers (42–44). DBS RNA profiling vs. whole venous blood RNA by genome-wide transcriptomics revealed the feasibility of DBS in field-based studies of gene expression (43, 44). However, no studies have explored this type of host markers in HIV-exposed children the way our study does. This could be an advantage since our method uses significantly smaller blood volumes (140 μl) per individual than required for other analyses. As an example, ELISAs normally require 200 μl of serum per sample (100 μl in duplicate) for the analysis of a single marker (40, 45). With our method, we were able to measure the expression of 12 genes related to inflammation and immune system uniquely using two spots from DBS (70 μl per spot). This reduction in sample volume is especially interesting in groups where sample extraction is more limited, such as the pediatric population.

Immune status monitoring could aid in predicting the risk of HIV-related comorbidities, chronic immune dysfunction, and especially persistent inflammation due to chronic HIV production, all of which contribute to the enhanced risk of morbidity and mortality among people with HIV (15, 18). In this context, this pilot study provides pioneering data on the expression of 12 immune markers in three groups of pediatric patients with or without HIV infection or exposure including IL-6, CD14, and CD163, which are immune biomarkers of myeloid origin with major clinical impact in terms of predicting morbidity (12, 31, 45, 46). Our data obtained by PCA and the single marker-by-marker analysis demonstrate that both HIV exposition during pregnancy and HIV infection alter the immune profile. The main limitation of this work is that it involves a pilot study that analyzes a small pediatric cohort. However, despite the low number of participants in each group, we were able to observe significant differences in the expression profiles of the immune biomarkers across groups, strongly suggesting the effect of HIV infection and/or HIV exposure on these immune biomarker's expression.

It is well-known that HIV infection increases plasma levels of some soluble immune biomarkers compared with uninfected (10, 39, 47, 48), even in well-controlled ART-treated HIV-infected with suppressed viremia (15, 39). We found that HIV+ children presented significantly higher levels of seven biomarkers (IL-6, CD14, HVEM, B7.1, Siglec-10, HIF-1α, and CD163) than the UU group. These results are in agreement with previous studies reporting an increase in soluble IL-6, CD14, and CD163 in plasma associated with HIV infection (12, 39, 48) and a higher CD163 level in HIV-infected macrophages due to chronic inflammation (40). CD163 has been independently associated with incidence of chronic kidney, lung, and liver disease in treated individuals with HIV (40). Regarding HIV exposure, our study reports pilot data that show significantly higher mRNA expression levels of seven immune biomarkers (IL-6, CD14, HVEM, B7.1, Siglec-10, HIF-1α, and IRAK-M) in HIV-infected and HIV-exposed children vs. UU. Only few studies have analyzed immune biomarkers (mainly soluble) in HEU, describing decreased T cell functionality after pathogenic stimuli (13) and higher inflammation and monocyte activation by increased levels of some soluble inflammatory biomarker in plasma (10, 11) of HEU adults compared with UU. Nonetheless, none have reported RNA levels of the 12 analyzed biomarkers in HEU compared with HIV+ and UU, as our study did, some of the latter never being reported in HEU or in HIV infection before (i.e., Siglec-10 or IRAK-M).

Interestingly, five biomarkers (CD14, HVEM, B7.1, Siglec-10, and HIF-1α) presented significantly higher expression in both HIV+ and HEU vs. UU, with HVEM and CD14 being significantly overexpressed among HIV+ vs. HEU. HVEM is highly expressed on HIV-infected T-CD4 cells and is involved at HIV entry (37), which might explain the significantly higher HVEM expression levels observed in HIV-infected compared with HEU and UU. However, the reason why HVEM appears to be greater expressed in the HEU vs. the UU group remains to be clarified. HIF-1α accumulates in the presence of HIV infection, since HIV-1 Vpr protein activates the oxidative stress pathway required for HIF-1α expression, explaining the high HIF-1α levels in HIV-infected individuals (38). Alternative molecular mechanisms for HIF-1α overexpression in HEU vs. UU should be elucidated, as occurs in other clinical contexts such as lung cancer (49).

Regarding B7.1, it is known that the HIV-1 nef protein removes B7.1 from the cell surface by modifying Golgi routes and preventing T lymphocyte activation (50). Siglec-10 was detected on subsets of human leukocytes, including eosinophils, monocytes, and a minor population of natural killer-like cells, and could function as an inhibitory receptor within the innate immune system (51). Nevertheless, given that both biomarkers were also significantly elevated in the HEU vs. UU group in the absence of viral proteins, the effect of HIV exposure in mRNA B7.1 and Siglec-10 expression needs to be further studied.

B7H5, PD-L1, and Galectin-9 levels remained similar across groups, suggesting a low effect of HIV presence or HIV exposure in their expression. Still, levels of the T cell activation suppressor and a novel costimulatory molecule regulating T cell responses in the B7 molecule family, B7H5, tended to be higher in the HIV group than in the HEU and UU groups and in the HEU vs. the UU group. This molecule has also been reported to be involved in cancer (52) but, to our knowledge, it has never been reported in the HIV field.

Another limitation to our research is the possible effect of antiretroviral exposure in our study population, since it dramatically reduces systemic inflammation and immune activation, although these do not decrease to levels comparable to the HIV-uninfected population (53), since residual immune activation, particularly monocyte/macrophage activation, were present (48). Moreover, the effect of each specific antiretroviral on inflammation and immune activation in HIV is still under research (53). However, due to the small sample size in our children cohort, a larger study in HIV-infected children with different therapeutic strategies should be performed to conclude the antiretroviral effects in immune system. Additionally, the lower age of HIV-exposed children vs. the remaining two groups was another limitation. However, the effect of children's age on immune marker expression remains unclear. Although some studies have observed a significant age-related increase in soluble CD14 and other biomarkers in HEU (21), others have only found significantly higher levels in HEU at birth, and the levels normalized after 6 months (11). We did not find any statistically significant correlation between age and any of the 12 studied immune markers in the studied cohort. These phenomena would reinforce the idea that the higher level of expression in some biomarkers vs. UU could be due to the HIV exposure and not due to the age. Therefore, the immune profile monitoring by immune biomarker quantification in blood should be considered to be integrated into PTMCT programs to monitor inflammation in HIV-infected newborns and in those uninfected but exposed children born to HIV-infected mothers.

Given that routine CD4/CD8 measurements were absent or scarce during the clinical follow-up of the children from Kinshasa under study, we provided the first results related to their immune status. Our results demonstrate that HIV exposure, and not only HIV infection, alters the level of some immune biomarkers in the pediatric study population. Furthermore, our study reveals some elevated biomarkers in children due to HIV exposure that can contribute to poor health outcomes related to chronic inflammation in HEU (9).

We have confirmed that DBS use for immune checkpoints monitoring could be an advantage in settings with limited infrastructure for blood processing or when low blood volume is available, as in neonates. Nevertheless, more studies comparing biomarkers' expression levels in paired DBS/plasma samples and in a larger population cohort are required. Development of simplified approaches for immune biomarker determinations at point of care is urgently needed for immune response monitoring in countries with limited infrastructures.

## Data Availability Statement

The original contributions presented in the study are included in the article/Supplementary Material, further inquiries can be directed to the corresponding authors.

## Ethics Statement

The studies involving human participants were reviewed and approved by La Paz Hospital Ethics Committee. Written informed consent to participate in this study was provided by the participants' legal guardian/next of kin.

## Author Contributions

AH and EL-C conceived and designed the study, contributed to data analysis, results discussion, and revised the paper and contributed to results discussion. AH, GR, and AN collaborated in sample shipping. AN selected clinical and epidemiological data from patients. MR-G and JA-O performed experimental assays and wrote the original manuscript. JA-O, MR-G, and CR performed the statistical analysis. All authors approved the final version.

## Conflict of Interest

The authors declare that the research was conducted in the absence of any commercial or financial relationships that could be construed as a potential conflict of interest.

## Supplementary Material

Figure S1 Correlation of age with the relative expression of CD14 **(A)**, HVEM **(B)**, IL-6 **(C)**, B7.1 **(D)**, Siglec10 **(E)**, HIF-1α **(F)**, CD163 **(G)**, IRAK-M **(H)**, B7H5 **(I)**, TNFα **(J)** PD-L1 **(K)**, and Galectin-9 **(L)** on mRNA extracted from DBS in a paediatric cohort of DRC. HIV+, HIV-infected children (red dots); HEU, HIV-exposed but born uninfected children (blue dots); UU, HIV-unexposed and uninfected children (green dots); P-values and Spearman R in Spearman correlation tests are shown.
