# Supplementary figures and images for: Dried Blood Specimens as an Alternative Specimen for Immune Response Monitoring During HIV Infection: A Proof of Concept and Simple Method in a Pediatric Cohort
Source: Front Med (Lausanne). 2021 Jun 15;8:678850. doi: 10.3389/fmed.2021.678850 (PMC8239183; doi:10.3389/fmed.2021.678850)

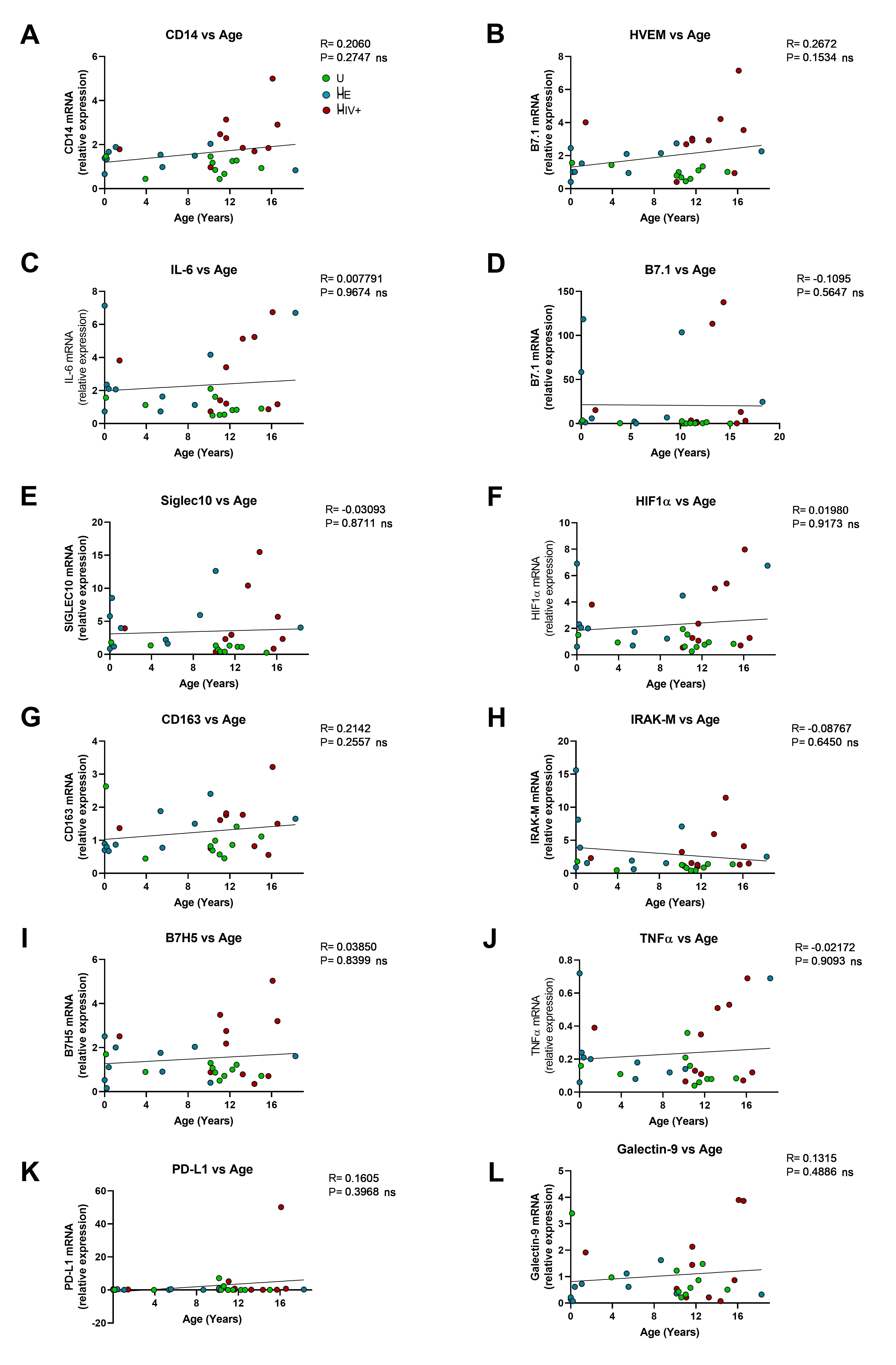

Supplement: Figure S1 — Correlation of age with the relative expression of CD14 (A), HVEM (B), IL-6 (C), B7.1 (D), Siglec10 (E), HIF-1α (F), CD163 (G), IRAK-M (H), B7H5 (I), TNFα (J) PD-L1 (K), and Galectin-9 (L) on mRNA extracted from DBS in a paediatric cohort of DRC. HIV+, HIV-infected children (red dots); HEU, HIV-exposed but born uninfected children (blue dots); UU, HIV-unexposed and uninfected children (green dots); P-values and Spearman R in Spearman correlation tests are shown. [file Image_1.jpg]
