# Supplementary material for: Dried Blood Specimens as an Alternative Specimen for Immune Response Monitoring During HIV Infection: A Proof of Concept and Simple Method in a Pediatric Cohort
Source: Front Med (Lausanne). 2021 Jun 15;8:678850. doi: 10.3389/fmed.2021.678850 (PMC8239183; doi:10.3389/fmed.2021.678850)
Supplement: Supplementary file 2 [file Table_1.docx]

**Supplementary Table S1. Sequence of primers used in RT-qPCR.**

| Gene | Forward primer (5’-3’) | Reverse primer(5’-3’) |
| --- | --- | --- |
| β-ACTIN | GTGGGGCGCCCCAGGCACCA | CTCCTTAATGTCACGCACGATTTC |
| B7.1 | GGGAAATGTCGCCTCTCTGA | TGTGGATTTAGTTTCACAGCTTGC |
| B7-H5 (VISTA) | CGTCCCTAGGTCCGGTGG | TGCGGTACCACGTCTTGTAG |
| CD14 | CTGCAACTTCTCCGAACCTC | TAGGTCCTCGAGCGTCAGTT |
| CD163 | CAGCGGCTTGCAGTTTCCTC | GGCCTCCTTTTCCATTCCAGAAA |
| HIF-1α | TTCCAGTTACGTTCCTTCGATCA | TTTGAGGACTTGCGCTTTCA |
| HVEM (TNFRSF14) | GTGCAGTCCAGGTTATCGTGT | CACTTGCTTAGGCCATTGAGG |
| IL-6 | ATTCGGTACATCCTCGAC | GGGGTGGTTATTGCATC |
| IRAK-M | TTTGAATGCAGCCAGTCTGA | GCATTGCTTATGGAGCCAAT |
| Galectin-9 (HUAT) | TCTGGGACTATTCAAGGAGGTC | CCATCTTCAAACCGAGGGTTG |
| PD-L1 (CD274) | TGGCATTTGCTGAACGCATTT | TGCAGCCAGGTCTAATTGTTTT |
| Siglec-10 | CCAAAAAGGCCAGTTCCTGC | GTGCCGTTCCCAAGGTTTTC |
| TNFα | GCCTCTTCTCTTTCCTGATCGT | CTCGGCAAAGTCGAGATAGTCG |
