# Supplementary material for: Dried Blood Specimens as an Alternative Specimen for Immune Response Monitoring During HIV Infection: A Proof of Concept and Simple Method in a Pediatric Cohort
Source: Front Med (Lausanne). 2021 Jun 15;8:678850. doi: 10.3389/fmed.2021.678850 (PMC8239183; doi:10.3389/fmed.2021.678850)
Supplement: Supplementary file 3 [file Data_Sheet_1.doc]

**DBS AS ALTERNATIVE SPECIMEN FOR IMMUNE RESPONSE MONITORING DURING HIV INFECTION: A PROOF OF CONCEPT AND SIMPLE METHOD IN A PAEDIATRIC COHORT**

Short title: HIV exposure and infection on immune profile in children

Marina RUBIO-GARRIDO^1,*^, José AVENDAÑO-ORTIZ^2,3,*^, AdolpheNDARABU^4^, Carolina RUBIO^3^, Gabriel REINA^5^, Eduardo LÓPEZ-COLLAZO^2, 3, #^, África HOLGUÍN ^1, #^

^1^ HIV-1 Molecular Epidemiology Laboratory, Microbiology Department. Ramón y Cajal University Hospital-IRYCIS and CIBERESP-RITIP, Madrid, Spain

^2^ Innate Immunity Group, IDIPaz-La Paz University Hospital, Madrid, Spain

^3^Tumor Immunology Laboratory and Innate Immunity Group, IDIPaz-La Paz University Hospital, Madrid, Spain

^4^Monkole Hospital, Kinshasa, Democratic Republic of Congo

^5^ University Clinic of Navarra, Pamplona, Spain

*These authors contributed equally to this work.

^#^Corresponding authors:

África Holguín

HIV-1 Molecular Epidemiology Laboratory

IRYCIS- Ramón y Cajal University Hospital-and CIBERESP-RITIP

Crta. Colmenar Viejo, Km 9,100, 28034 Madrid, Spain

E-mail: [africa.holguin@salud.madrid.org](mailto:africa.holguin@salud.madrid.org)

Phone: +34-913368153

Eduardo López-Collazo

IdiPAZ, La Paz University Hospital,

Paseo de La Castellana 261, 28046 Madrid, Spain

E-mail: [elopezc@salud.madrid.org](mailto:elopezc@salud.madrid.org)

Phone: +34-912071029

**STROBE Statement**

Checklist of items that should be included in reports of observational studies.

| **Section/Topic** | | | Item No | | | Recommendation | | | Reported on Page No | |
| --- | --- | --- | --- | --- | --- | --- | --- | --- | --- | --- |
| Title | | 1 | | | EFFECT OF HIV EXPOSURE ON IMMUNE PROFILE: A PILOT STUDY ON A PAEDIATRIC POPULATION | | | 1 | | |
| Introduction | | | | | | | | | | |
| Background/rationale | | | 2 | | | The implementation of programs to prevent mother-to-child HIV transmission reduces the number of newly infected children but not of infants exposed during pregnancy and breastfeeding. HIV exposed but uninfected children (HEU) present higher risk of morbidity, mortality and growth failure rate than HIV unexposed an uninfected children (UU). Immune biomarkers analysis could improve the study of HIV disease progression, predicting comorbidity risk. | | | 4-5 | |
| Objectives | | | 3 | | | The main objective was to evaluate the usefulness of DBS samples as an alternative to the sample or serum. In addition, we analyzed the influence of HIV infection and exposure-HIV on the immune status of three groups of children (HIV+, HEU and UU). | | | 6 | |
| Methods | | | | | | | | | | |
| Study design | | | 4 | | | Pilot case-control study which measures gene expression levels of 12 immune biomarkers (CD14, HVEM, B7.1, HIF-1α, Siglec 10, IRAK-M, CD163, B7H5, PD-L1, IL6, TNFα and Galectin-9) in DBS collected from April to November 2016 from 30 paediatric participants: 10 HIV infected, 10 HEU and 10 UU. | | | 7 | |
| Setting | | | 5 | | | Paediatric participants were under clinical follow-up in Pediatric Unit at Monkole Hospital in Kinshasa (DRC). | | | 7 | |
| Participants/study size | | | 6 | | | Thirty children: 10 HIV infected, 10 HIV exposed but uninfected and 10 HIV unexposed and uninfected children. | | | 7 | |
| Variables | | | 7 | | | HIV infection, HIV exposition, level of gene expression of 12 inflammatory biomarkers: CD14, HVEM, B7.1, HIF-1α, Siglec 10, IRAK-M, CD163, B7H5, PD-L1 IL6, TNFα, and Galectin-9, some of them never reported before in HEU or in HIV infection (i.e. Siglec-10 or IRAK-M). | | | 8 | |
| Data sources/measurement | | | 8* | | | Gene expression levels of these 12 immune biomarkers were quantified using RT-qPCR assay on mRNA extracted from DBS. Actin was used as a housekeeping gene to measure the relative expression of each biomarker.  Positive serological HIV status by Bio-Rad Geenius HIV 1/2 Confirmatory Assay and confirmed by Cepheid Xpert HIV-1 Qual.  HIV-1 viremia was quantified using COBAS® AmpliPrep/COBAS® TaqMan® HIV-1 Test v2.0 (Roche)  Antiretroviral exposure after birth in children and mother data according to clinical files | | | 8-9 | |
| Quantitative variables | | | 9 | | | Gene expression levels of 12 immune biomarkers previously mentioned. | | | 9 | |
| Statistical methods | | | 10 | | | Principal components and Kruskal-Wallis non-parametric test was performed for overall comparison of the three groups. After that, the Mann–Whitney U test was used to study the differences among groups. The significance level used for the hypothesis contrast tests was set at p<0.05. Statistical analyses were conducted using Prism 6.0 software from GraphPad | | | 9 | |
| Results | | | | | | | | | |  |
| Participants | 11 | | | A total of 30 children: 10 HIV infected (HIV+), 10 HIV exposed but uninfected (HEU) and 10 HIV unexposed and uninfected (UU) children | | | 10 | | |  |
| Descriptive data | 12 | | | Descriptive data from the groups of individuals are summarised in Table 1 of the manuscript. | | | 10 | | |  |
| **Section/Topic** | Item No | | | Recommendation | | | Reported on Page No | | |  |
| Main results | 13 | | | The results of PCA showed that the immune marker profiles in UU children differed substantially from those HEU and HIV-infected groups. Once the differences in the immunological profiles between the groups were proven, we decided to study the expression profile of each marker separately. HIV+ children presented significantly higher levels of seven biomarkers (CD14, IL-6 HVEM, B7.1, Siglec-10, HIF-1α and CD163) than UU group. In HEU, we found seven biomarkers significantly elevated (CD14, IL-6, HVEM, B7.1, Siglec-10, HIF-1α and IRAK-M) *vs*. UU. Six biomarkers (CD14, IL-6, HVEM, B7.1, Siglec-10 and HIF-1α) showed significantly higher expression in both HIV+ and HEU *vs.* UU, with HVEM and CD14 significantly overexpressed among HIV+ *vs.* HEU. The strength of our pilot study is that we present data on gene expression level of 12 immune checkpoints molecules, some of them never before reported in HEU or in HIV infection (i.e. Siglec-10 or IRAK-M).  Given routine CD4/CD8 measurements were absent or scarce during the clinical follow-up of the children from Kinshasa under study, we provide the first results related to their immune status. Our results demonstrate that HIV exposure, and not only HIV infection, alters the level of some immune biomarkers in the paediatric study population. | | | 9-13 | | |  |
| Discussion | | | | | | | | | |  |
| Key results | 14 | | | This pilot study provides pilot data on the expression of 12 immune markers in three groups of paediatric patients with or without HIV infection or exposure including CD14 and CD163, which are immune biomarkers of myeloid origin with the greatest clinical impact in terms of predicting morbidity and mortality. No previous study has reported RNA levels of the 12 analysed biomarkers in HEU compared with HIV+ and UU, as our study did, some of them never before reported in HEU or in HIV infection (i.e. Siglec-10 or IRAK-M). Our data by PCA and the single marker-by-marker analysis demonstrates that both HIV exposition during pregnancy and HIV infection alters the immune profile. We observed significant differences in the expression of specific biomarkers across groups, reporting new data on the effect of HIV infection and/or HIV exposure on the immune profile in children. It is also the first study using dried blood instead of plasma/serum to measure the gene expression of these specific immune markers, supporting DBS use as alternative sample to plasma to quantify immune status. DBS use for immune immunocheckpoints monitoring could be an advantage in settings with limited infrastructure for blood processing or when low blood volume in available, as in neonates. | | | 15-16 | | |  |
| Limitations | 15 | | | Being a pilot study, the sample size is limited. It is also important to consider the difference in age among the groups of children. Another limitation is the possible effect of ARV in the treated children. This comparison could be a very interesting way to definitively validate DBS as an alternative sampling method in the future. More studies are required comparing biomarker´s expression levels in paired DBS/plasma samples and in larger population sets. | | | 17-18 | | |  |
| Interpretation | 16 | | | Since immune status monitoring could also predict the risk of HIV-related comorbidities and chronic immune dysfunction, our findings suggest that immune profile monitoring by immune biomarker quantification in blood should be integrated into PTMCT programs to monitor inflammation in HIV infected newborns as well as in those uninfected but exposed children born to HIV-infected mothers. | | | 18 | | |  |
| Generalisability | 17 | | | Studies using large data sets are needed to test the generalizability of our results | | | 19 | | |  |
| Other Information | | | | | | | | | |  |
| Funding | 18 | | | This study has been funded by Instituto de Salud Carlos III through the projects “PI14/01234, PIE15/00065, PI18/00148 and PI18/00904” (Co-funded by European Regional Development Fund, "A way to achieve Europe") and by Fundación Familia Alonso. JAO and MRG was supported by grants from Comunidad de Madrid (PEJ15/BIO/AI-0021 and PEJD-2017/PRE-BMD-4497, respectively). | | | 19 | | |  |
